# Functional accessory limb arising from the buttock: a case report

**DOI:** 10.1186/s12887-025-05712-7

**Published:** 2025-05-06

**Authors:** Mohammad Mahdi Ebrahiminasab, Taghi Baghdadi, Pouya Tabatabaei Irani, Soroush Baghdadi

**Affiliations:** 1 Joint Reconstruction Research Center, Tehran University of Medical Sciences, Tehran, Iran; 2 Department of Orthopaedic Surgery, University of California Los Angeles, 1225 15th St, Suite 2100, Los Angeles, Santa Monica, CA 90404 USA; 3 Orthopaedic Institute for Children, Los Angeles, USA

**Keywords:** Polymyelia, Accessory limb, Congenital anomaly, Limb differences

## Abstract

**Background:**

Polymelia, or accessory limb, is a rare congenital abnormality with unknown etiology. Here we report the case of an infant with an accessory limb, review the diagnostic and therapeutic consideration of such cases, and highlight the potential challenges in management of an accessory limb.

**Case presentation:**

We report the case of a 2-month-old male with an accessory limb arising from the left buttock. The limb was sensate and mobile joints. Our diagnostic workup did not reveal associated spinal dysraphism, but a left dislocated hip was found. The limb was surgically removed, and the hip dislocation was eventually treated with open reduction, with excellent results.

**Conclusion:**

This study reports a rare case of accessory limb and reviews the important considerations in the management of this condition.

**Clinical trial number:**

Not applicable.

## Background

Polymelia, or accessory limb, is an extremely rare congenital abnormality. This condition is defined as the presence of supernumerary limb segments, often associated with spina bifida and other congenital anomalies [1–3]. Polymelia is further classified as cephalomelia (extralimb on the head), thoracomelia (extralimb on the thorax), notomelia (extralimb on the back), and pyromelia (extralimb arising from the pelvis). The etiology is not fully known, but intrauterine injuries and exposure to medications and toxins have been proposed [2]. Chromosomal and genetic abnormalities have also been reported in association with polymyelia [1, 4]. Here, we report a rare case of male child born with a functioning accessory limb arising from the pelvis (pyromelia) associated with an ipsilateral developmental hip dislocation. We also review the literature, the common presentations and associated pathologies, and treatment options.

## Case presentation

**Fig. 1:**
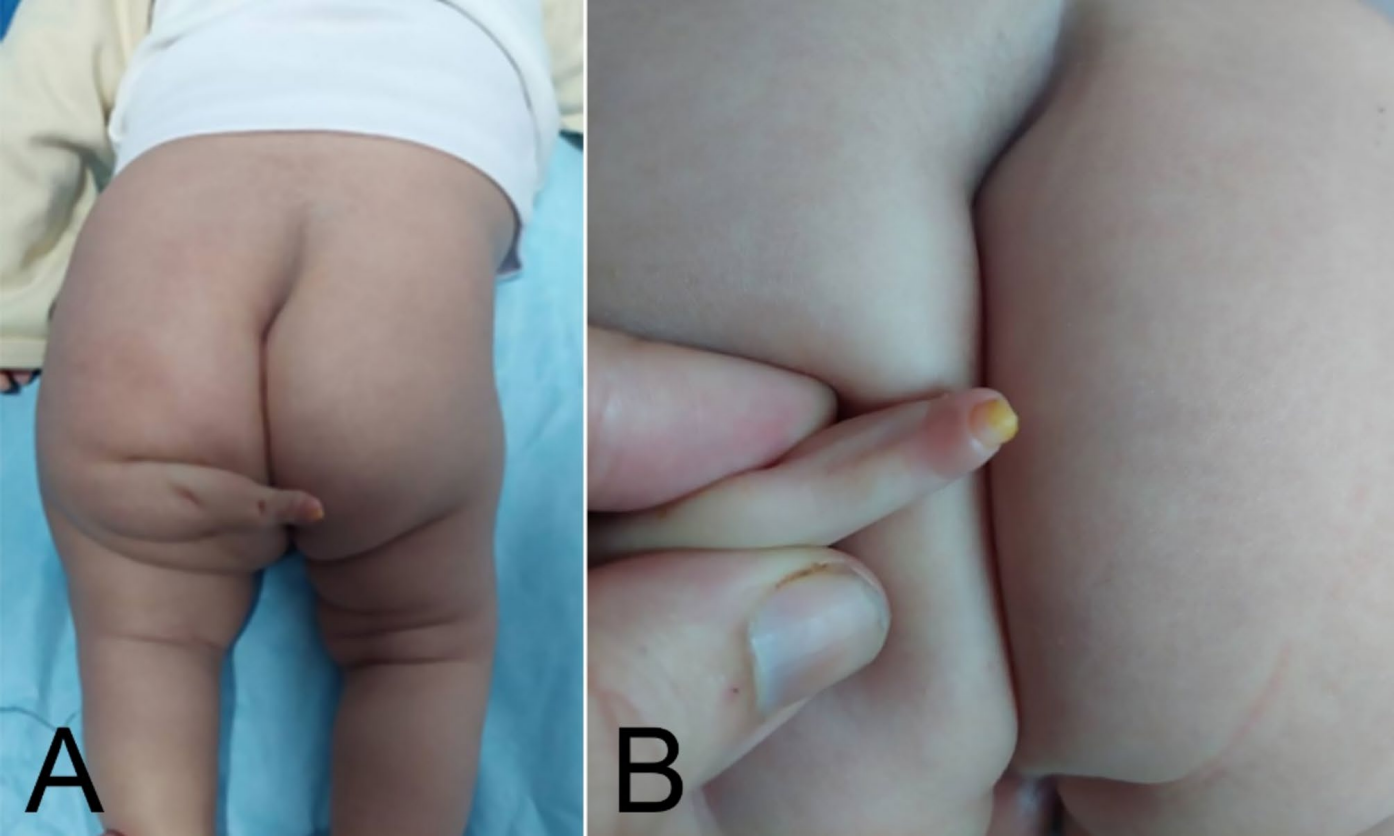
Posterior view of the patient’s accessory limb arising from the left buttock (**A**). Magnified view of the limb shows skin texture and nail at distal end of the limb **(B).**

**Fig. 2:**
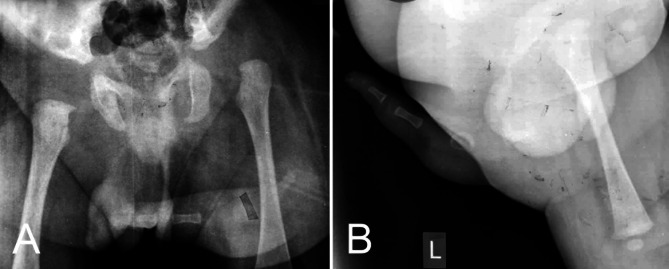
Pelvic X-ray with anteroposterior (**A**) and lateral (**B**) views of the accessory limb. The limb does not appear to be articulating with the pelvis. Note the left dislocated hip

A 2-month-old male infant was visited at our clinic having an underdeveloped accessory limb arising from his left buttock. Patient was born via vaginal delivery at 38 weeks gestation to a 27-year-old mother. The mother did not receive an ultrasound during pregnancy, and this condition was diagnosed at the time of birth. The pregnancy and delivery were uneventful. Parents were healthy and non-consanguineous. On examination, the accessory limb was arising from the left ischial region, had 1 finger with 2 joints with near normal skin texture (Fig. 1). Neurological examination showed preserved sensation and motor function having active flexion and extension. Deep tendon reflexes were not elicited in the accessory limb, but the joints had 0–60 degrees of passive motion. The lower limbs were normal in shape and function; however, the left hip had a positive Ortolani test with limited abduction. No other skeletal, urogenital or cardiac anomalies were found on clinical and ultrasound exams. Karyotyping was performed which was normal. X-ray examination confirmed a left hip dislocation with the accessory limb having 3 underdeveloped bones with no articulation with the pelvis (Fig. 2). An ultrasound was obtained, showing no articulation with the pelvis and one neurovascular bundle.

**Fig. 3:**
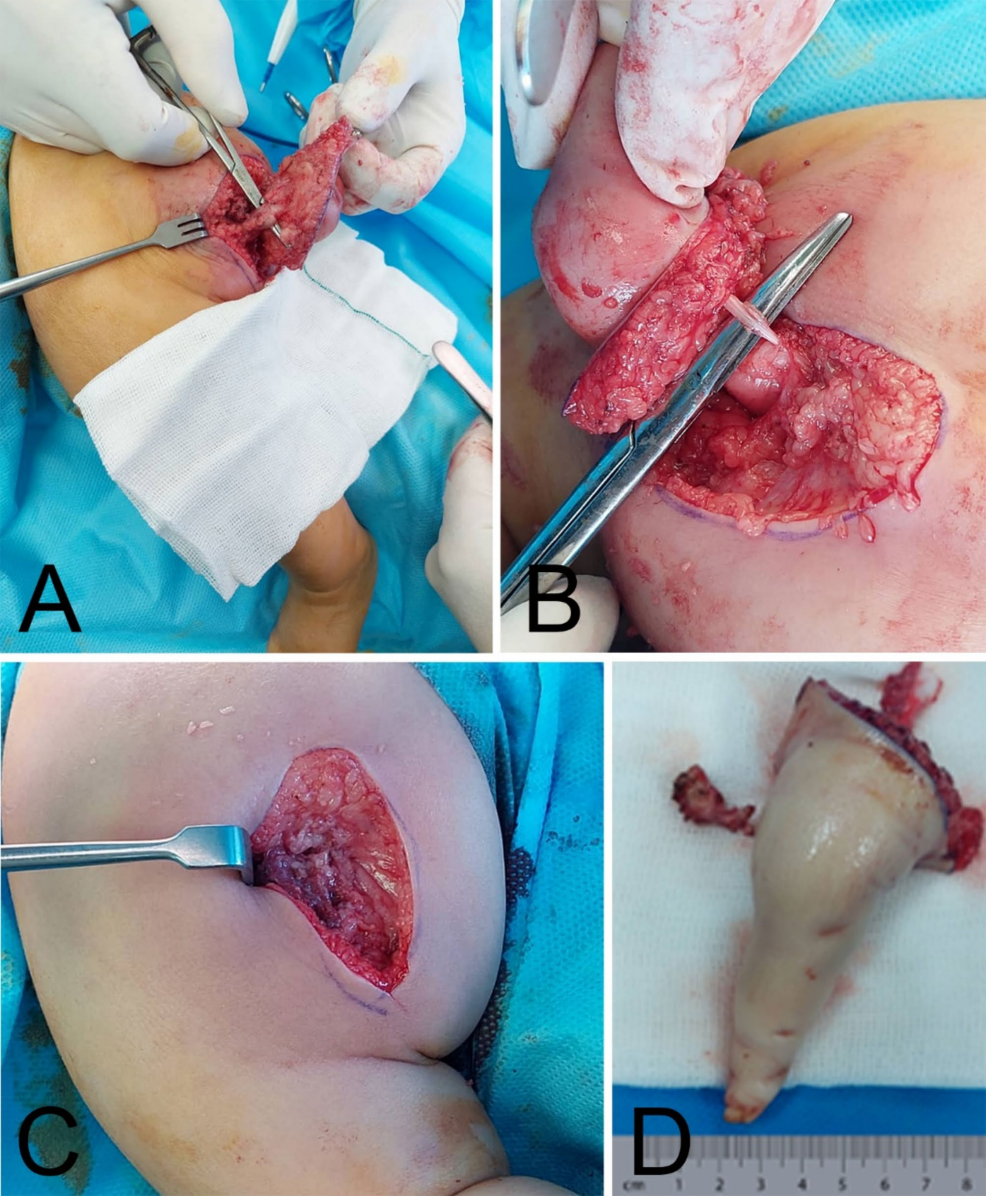
With the patient in the lateral position (**A**), an elliptical incision was made around the accessory limb. At the base of the incision, the neurovascular bundle was identified (**B**) and tied at the most proximal point. Post-excision clinical picture (**C**) and the gross examination of the excised limb (**D**) are shown

After diagnostic workups, the child underwent exploration and removal of the accessory limb. An elliptical incision was made around the skin of the extra limb. The neurovascular bundle arising from the pelvis was ligated and the accessory limb fully excised at its origin (Fig. 3).

Upon gross anatomic examination of the excised limb, there was the appearance of an underdeveloped digit with only one toe having a nail. Several other underdeveloped bones were observed when the limb was dissected, including the appearance of metatarsal, tarsal, leg bones accompanied by a small femoral attachment.

On microscopic evaluation, the accessory limb consisted of underdeveloped bony and cartilaginous structures surrounded by fat, muscles and blood vessels. However, abnormal neuroglial or excessive tissue such as hamartoma or teratoma were not seen.

**Fig. 4:**
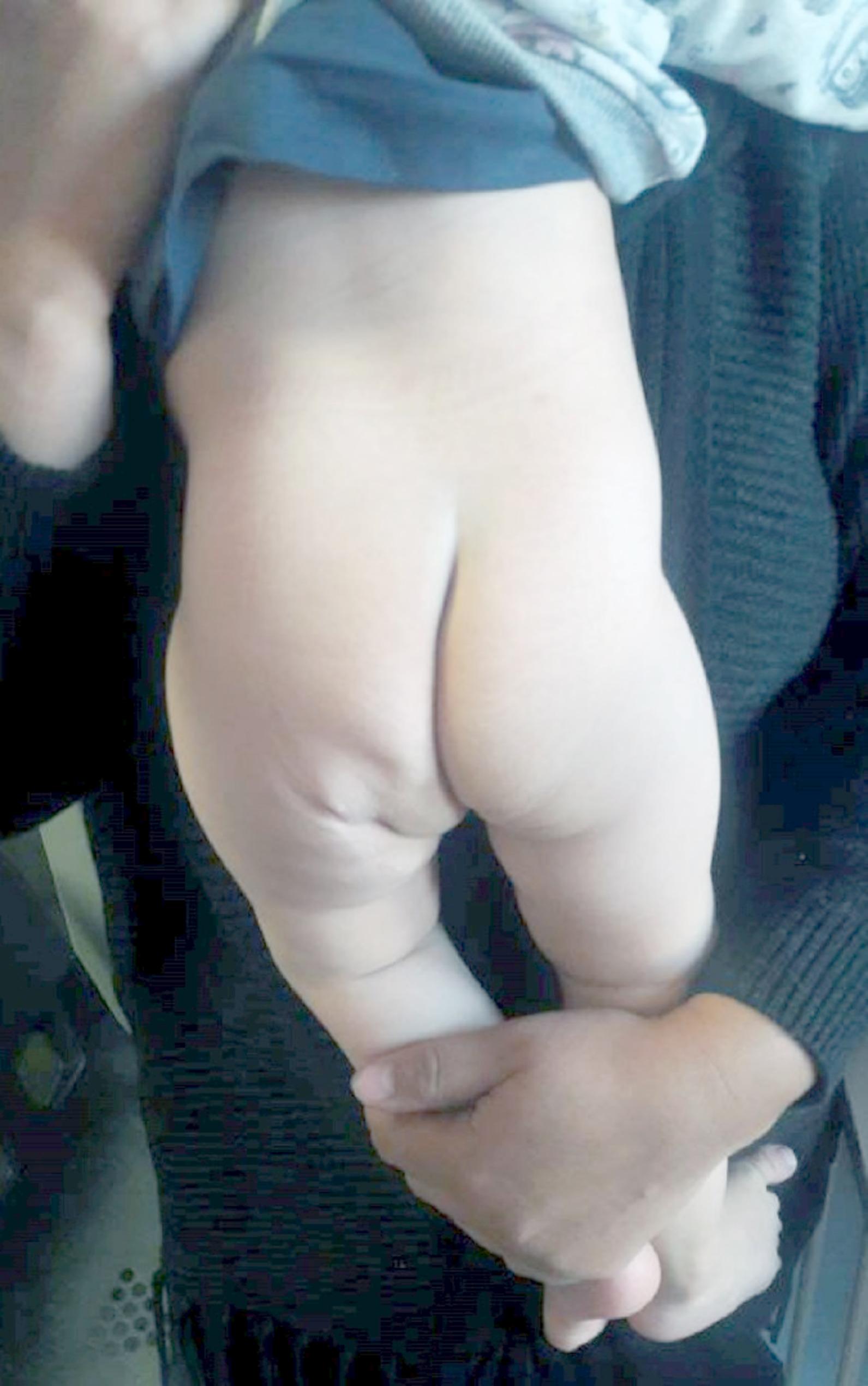
Clinical photograph of the patient’s back 18 months after resection, showing minimal scarring and satisfactory remodeling of excessive tissue

**Fig. 5:**
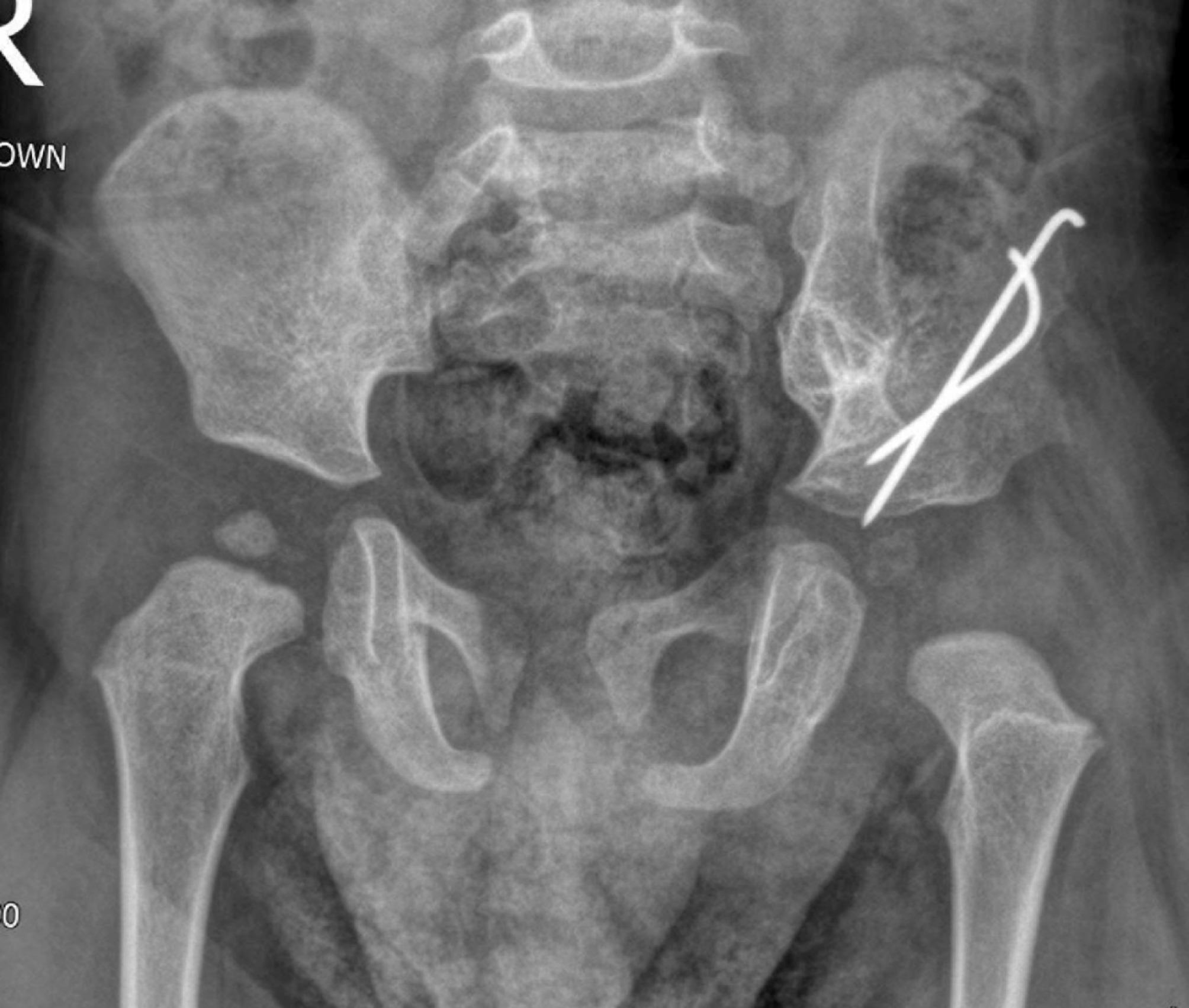
Anteroposterior pelvic radiograph 18 months after initial resection and 4 months after open reduction of the left hip, showing reduction of the hip

The patient had an uneventful recovery. Pavlik harness treatment was initiated for the left hip dislocation, which failed to reduce the hip. The patient was eventually treated with open reduction of the hip and innominate osteotomy at the age of 14 months. At the latest follow-up, 18 months after the initial accessory limb resection, the clinical (Fig. 4) and pelvic radiograph (Fig. 5) showed excellent results with a reduced hip.

## Discussion

Accessory limb is an extremely rare congenital anomaly, and only a few documented cases have been reported. There is no uniform terminology available, and this condition is referred to as tripedus, polymyelia, heteropegus, rachypagus, dysraphic appendages, heterotopic redundancy, aborted twinning, aborted or rudimentary accessory limb, caudal duplication and leg duplication in the literature [1, 5–8]. The definition based on the attachment site, i.e. cephalomelia, thoracomelia, notomelia, and pyromelia seems to be simple and easy to use, and may have clinical and therapeutic implications [2].

The first report of polymelia was published in 1889 by Jones et al., reporting on a baby born with an extra limb arising from the thoracic region [9]. Congenital accessory limbs are frequently associated with spinal and other skeletal anomalies [1–4, 10–13]. The published literature suggests a female preponderance of 4:1 [1, 2, 12]. The patient presented in this report was male and had an associated left developmental dysplasia of the hip.

While the accessory limb in this case was arising from the ischial region, corresponding to a pyromelia anomaly, different attachment sites have been reported. Early splitting of the limb bud may cause an accessory limb to be arising from the lumbosacral region accompanied by spinal dysraphism. However, if the limb bud splitting occurs later in the embryonic life, the extra limb would arise more distally including the acetabulum, buttock or thigh rather than the back or lumbosacral region. The literature seems to support the observation that the later development of an accessory limb in utero, and therefore an appendicular rather than axial attachment is less frequently associated with spinal dysraphism [2, 4, 12, 15].

The authors would like to emphasize the importance of antenatal diagnosis. The family did not receive proper pregnancy care including ultrasound. This not only adds to the psychological burden to parents, but it also delays care, including diagnostic workups and treatment.

From a diagnostic standpoint, there are reports of teratomas initially believed to be an accessory limb, given the presence of developed mesodermal tissue in a teratoma. This distinction is extremely important, since a teratoma needs to be excised in its entirety to prevent future growth, but the base of an accessory limb may be retained for an improved cosmetic or functional outcome. In a report by Unterscheider et al., an accessory limb with syndactyly was found to be a teratoma on microscopic evaluation [14]. Similarly, in the present case, microscopic evaluation of the excised accessory limb, provided no evidence indicating that this structure was a teratoma. Therefore, a thorough pre-operative workup is recommended, which may include ultrasound and MRI examinations to rule out associated abnormalities and the presence of a teratoma.

The current case highlights several considerations when excising accessory limbs. First, a review of the literature shows that these are often highly developed appendages, which necessitates an understanding of the nervous and vascular supply to the limb. A doppler ultrasound, CT with and without contrast, and MRI are invaluable in pre-operative planning. Skin incisions should be kept as distal as possible in case more coverage is needed. Tendons could be cut sharply, but nerves should be followed as proximal as possible and tied to prevent neuroma formation. Finally, remodeling potential should not be overlooked, and a fairly prominent stump could be unnoticeable with atrophy and remodeling. Therefore, under-resection is preferred to over-resection.

## Conclusion

Accessory limbs are rare congenital anomalies that are a mental and emotional burden to the family if not treated early. Diagnostic workup focuses on evaluating associated pathologies and the neurovasculature of the limb. While the case presented here did not have spinal dysraphism, a dislocated hip on the ipsilateral side was diagnosed and treated with an open reduction and pelvic osteotomy. This case highlights the important pre-operative and technical considerations with accessory limbs.

## Data Availability

All data generated or analyzed during this study are included in this published article.

## References

[1] Park K-B, Kim Y-M, Park J-Y, Chung M-L, Jung Y-J, Nam S-H. An accessory limb with an imperforate anus. Ann Surg Treat Res. 2014;87(4):213–6. 25317418 10.4174/astr.2014.87.4.213 PMC4196430

[2] Verma S, Khanna M, Tripathi V, Yadav N. Occurrence of polymelia in a female child. J Clin Imaging Sci. 2013;3. 10.4103/2156-7514.111235 PMC3690670 23814690

[3] Bayri Y, Tanrıkulu B, Ekşi MŞ, Dağçınar A. Accessory lower limb associated with spina bifida: case report. Childs Nerv Syst. 2014;30:2123–6. 25092402 10.1007/s00381-014-2475-7

[4] Mohindra S, Batish A, Patil NR, Tripathi M. Fully developed accessory lower limb with tethered cord: are these two anomalies related? Childs Nerv Syst. 2021;37:325–8. 32328705 10.1007/s00381-020-04616-4

[5] Sarris CE, Tomei KL, Carmel PW, Gandhi CD. Lipomyelomeningocele: pathology, treatment, and outcomes: A review. Neurosurg Focus. 2012;33(4):E3. 23025444 10.3171/2012.7.FOCUS12224

[6] Priyawansha Y, Munasinghe K, Manike N. A rare case of heterophagus twinning. Sri Lanka J Child Health. 2018;47(3).

[7] Bodeliwala S, Singh D, Singh H, Iqbal M, Agarwal A, Khurana P. Spinal dysraphism with tripedus: A child with three legs. Neurol India. 2017;65(1):214. 28084286 10.4103/0028-3886.198209

[8] Ijaz L, NADEEM MM. Vestigial accessory limbs with spina bifida: our 5–year experience. Pakistan J Neurol Surg. 2016;20(3):187–91.

[9] Jones R, Larkin FC. Removal of accessory limb and meningocele from the back of a child, and its anatomy. Br Med J. 1889;2(1493):310. 20752778 10.1136/bmj.2.1493.310 PMC2155450

[10] Chadha R, Bagga D, Malhotra C, Dhar A, Kumar A. Accessory limb attached to the back. J Ped Surg. 1993;28(12):1615–7. 10.1016/0022-3468(93)90118-5 8301511

[11] Nanni L, Perrelli L, Velardi F. Accessory lower limb in a newborn with multiple malformations. Eur J Pediatr Surg. 1994;4(01):51–3. 8199136 10.1055/s-2008-1066068

[12] Saaiq M, Zimri FK, Zaman K-u. Successful treatment of well-developed accessory lower limb associated with spinal dysraphism. World J Plast Surg. 2020;9(1):73. 32190596 10.29252/wjps.9.1.73 PMC7068195

[13] Taniguchi K. Baby with a third leg. 1975. 10.1016/s0022-3468(75)80025-x 1117388

[14] Unterscheider J, O’Byrne J, Foran A, Robinson I, Ryan S, Devaney D, et al. Prenatal identification of an accessory lower limb. Prenat Diag. 2011;31(12):1203–4. 10.1002/pd.2846 21898470

[15] Sharma L, Singh R, Bhargava J, Sharma V. Accessory limbs with spinal lesions. Pediatr Surg Int. 1991;6:227–9.

